# Evaluating the Capability of Grass Swale for the Rainfall Runoff Reduction from an Urban Parking Lot, Seoul, Korea

**DOI:** 10.3390/ijerph15030537

**Published:** 2018-03-16

**Authors:** Muhammad Shafique, Reeho Kim, Kwon Kyung-Ho

**Affiliations:** 1 Department of Smart City and Construction Engineering, Korea Institute of Civil Engineering and Building Technology, University of Science and Technology (UST), 217, Gajeong-ro, Yuseong-gu, Daejeon 34113, Korea; shafique@ust.ac.kr; 2 Environmental & Plant Engineering Research Institute, Korea Institute of Civil Engineering and Building Technology, 83, Goyangdae-ro, Ilsanseo-gu, Goyang-si, Gyeonggi-do 10223, Korea; 3 Urban Water Cycle Research Center, Korea Institute of Safe Drinking Water Research, Anyang si, Gyeonggi-do 14059, Korea; kwonkh@kisd.re.kr

**Keywords:** grass swale, runoff, stormwater management, soil moisture, rain events

## Abstract

This field study elaborates the role of grass swale in the management of stormwater in an urban parking lot. Grass swale was constructed by using different vegetations and local soil media in the parking lot of Mapu-gu Seoul, Korea. In this study, rainfall runoff was first retained in soil and the vegetation layers of the grass swale, and then infiltrated rainwater was collected with the help of underground perforated pipe, and passed to an underground storage trench. In this way, grass swale detained a large amount of rainwater for a longer period of time and delayed peak discharge. In this field study, various real storm events were monitored and the research results were analyzed to evaluate the performance of grass swale for managing rainfall runoff in an urban area. From the analysis of field experiments, grass swale showed the significant rainfall runoff retention in different rain events. Grass swale markedly reduced total rainfall runoff volume and peak flow during the small storm events of intensity about 30 mm/h. From the analysis, on average rainfall runoff retention from the grass swale was found around 40 to 75% during the various small rain events. From the results, we can say that grass swale is a stormwater mitigation practice which can help avoid flash flooding problems in urban areas.

## 1. Introduction 

For the last few decades, rapid urbanization is the main cause of surface water deterioration around the globe [1,2]. In urban areas, the impervious surface area is continuously increasing, which in turn increases the rainfall runoff volume, intensity and peak flow. As a result of this more runoff is collected on the surface, which causes flash flooding and water quality degradation problems [2,3,4,5,6,7]. To encounter these adverse urban water-related problems associated with urban development, Low Impact Development (LID) practices have been applied in different countries and shown multiple benefits for stormwater management in urban areas [4,5,6,7,8,9,10]. Within this concept infiltration practices such as swales and rain gardens are the most commonly used LID practices [4,5]. Infiltration practices are designed to contribute to urban drainage by retaining a large amount of rainfall runoff and thus reducing the flow volume and peak flow which reduces the chances of flash flooding in urban areas. 

Grass swales are vegetated open channels usually designed to convey runoff, reduce rainfall runoff by infiltration and boost the runoff quality from filtration through the soil media [11,12,13,14,15]. Grass swale uses different kind of vegetation, which can infiltrate the surface water into the underground soil [12,15,16]. Studies have shown that grass swale can be used as a stormwater mitigation strategy which reduces the pressure on the existing sewer system by retaining a large amount of rainfall runoff [12,17]. 

Previous studies [18,19,20,21,22,23,24,25] in different countries where the grass swale system was applied at different locations under cold climatic conditions (i.e., Sweden, USA and Germany) have indicated that grass swales are very efficient systems for stormwater management, however, there is still a need to design a more efficient swale system for storm water management in Asian countries like South Korea or China. For this purpose, this study was designed to check the performance of grass swale for stormwater management in Seoul, Korea.

To date, several studies have shown the grass swale performance for the rainfall runoff delay and volume reduction in urban areas [16,17,18,19,20]. Deletic and Fletcher [18] evaluated the performance of the grass swale system in Brisbane (Australia) through field experiments. From the analysis, the research results indicated that grass swale reduced the rainfall runoff volumes 33–87% in different storm events. Similarly, Ackerman and Stein [19] reported results that demonstrated that the grass swale system reduced mean rainfall runoff volume by almost 52.5%. Barrett [20] studied a vegetated swale which contained permeable soil with low moisture content. He concluded that the swale has the ability to infiltrate up to 50% of the rainfall runoff volume and thus contribute to sustainable stormwater management in urban areas. Table 1 lists some of the volume reductions achieved by using grass swale at different locations. 

Fassman and Liao [21] performed field experiments in New Zealand to check the hydrological performance of grass swale. From the results, it was concluded that the grass swale reduced approximately 63% of the total runoff volume during different storm events. Similar procedures were followed by Rushton [22] and Deletic [23] to capture the runoff by swales and results showed that grass swale can capture 30% and 45.7% of the rainfall runoff, respectively. Rujner et al. [24] performed a field experiment on the 30-m long grass swale located in Luleå, Sweden. They analyzed 14 rainfall events to check the performance of swale for runoff reduction and it was concluded that the swale system reduced the total runoff volume by 40% to 55%. Davis et al. [25] studied the highway grass swale for the stormwater management; the results allow them to conclude that the grass swale can reduce up to 59% of the total runoff during different rain events. Increased rainfall runoff retention time and reduction of the outflow volume through grass swale showed that grass swale has the potential to manage the stormwater near the site [4,21,24,25,26,27,28]. Infiltration and evapotranspiration losses are the two main causes of the volume reduction by the grass swale [21,24]. The minimum criteria that should be followed for the grass swale infiltration rate is 2.7 mm/h [26]. Soil moisture content is also a very important factor to catch more rainfall during different storm events [20,29], however until now there has been a lack of studies that show how soil moisture contributes to the stormwater management in urban areas. The low soil moisture of the soil is a very important factor to capture more rainfall runoff [24]. 

The above global literature provides grass swale hydrological performance and a knowledge gap to enhance the grass swale stormwater management performance in Korean conditions. After promulgation of the Special Law on Waterfront Development for Low Impact Development (LID) applications [4], the Korean government had started active work on the LID projects to make cities more sustainable and more resilient to the effects of climate change. The Korean government has adopted the concept of water cycle cities [4,30] for application of new innovative design LID practices such as green roofs, blue roofs, rain gardens, grass swales [31,32,33,34,35,36,37], etc. in Korea. In Korea, the term LID is also known as Healthy Water Cycle City, Rain City and Green City [4,31]. The main idea behind this study was to retrieve the natural hydrological conditions of urban areas. Grass swale application in this experiment was also the part of that project. Until now, there have been very few studies [27,37] done to evaluate the performance of the grass swale system for stormwater management in Korea. Therefore, this field study was carried out to check the grass swale performance for stormwater management in a highly urbanized area of Seoul. This study also investigated the rainfall runoff retention performance of grass swale under real storm event conditions. 

## 2. Study Objectives

The main purpose of this experiment was to calculate the overall hydrological performance of grass swale to reduce the risk of flash flooding in urban areas. This study examined the overall runoff reduction capability of grass swale in real storm events. Overall objectives of this research study were listed below:

■ To measure the soil water contents which significantly influence the rainfall runoff reduction.
■ Investigate the performance of grass swale to detain rainfall runoff in different precipitation events. Evaluate the feasibility of grass swale as a Low Impact Development (LID) measure to reduce the risk of urban flooding in Korea.

## 3. Materials and Methods

### 3.1. Study Area Descriptions and Details

**Figure 1:**
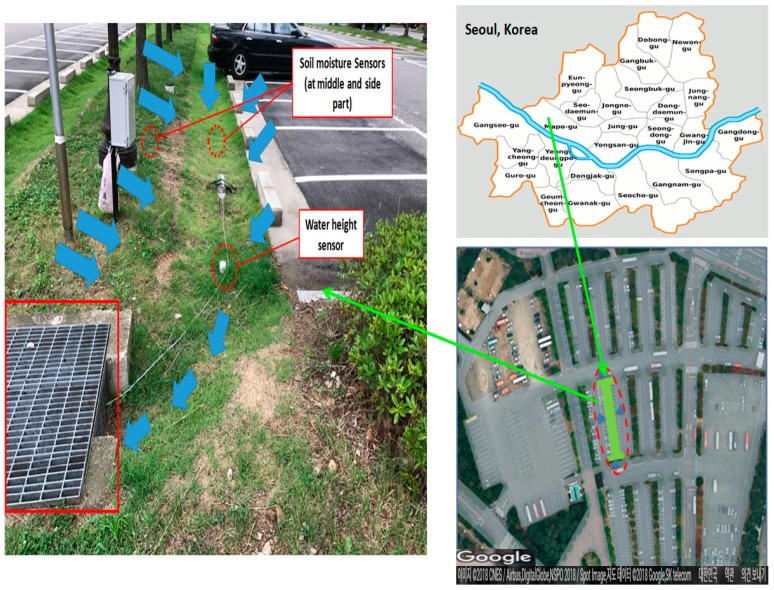
Diagram of the grass swale location and details.

This study was carried out on a grass swale system located in the Mapu-gu World Cup stadium, (Seoul, Korea, 37°33′15.77” N 126°54′33.34” E). In urban areas, due to the already built infrastructure, there is a very little space available to apply LID practices [5]. Generally, the optimum places where more available space for the application of LID practices exists are parks, school buildings and big parking lots, as compared to congested urban areas [5]. Therefore, this grass swale system was also constructed in an urban parking lot of Seoul, Korea, as shown in Figure 1. In the parking lot, multiple swale systems were constructed, however, we selected one grass swale system to perform our experiments, as marked in Figure 1. The main purpose for constructing these swale systems was to capture the surrounding rainfall runoff and retain and infiltrate the water into the ground to reduce the flash flooding problems in that area. Figure 1 also shows the grass swale where the experiment was carried out and results were analyzed during different rain events. The dimensions of the selected swale system were 51 m long and 1.2 m wide. This swale system allows surrounding rainfall runoff from all sides to enter into it and detain it for a longer period of time and slowly infiltrate it into the ground surface. The water is first retained in the vegetation and soil media and then the infiltrated water is collected and infiltrated in an underground perforated pipe. Later on, remaining water from the perforated pipe is passed to an underground storage trench where water is stored and slowly infiltrated into the underlying soil. In this way more water is collected and distributed within the grass swale area. Consequently, less rainwater runoff collects on the surface, and there is reduced the risk of flash flooding problems in the urban area. Water height and soil moisture sensors were installed at the different positions in the grass swale to check water height and soil moisture values in different rain events. Soil moisture values were collected at two points in the grass swale system. Figure 1 also shows that this swale system has a slope and all the water enters at the marked point shown on the left side. 

**Figure 2:**
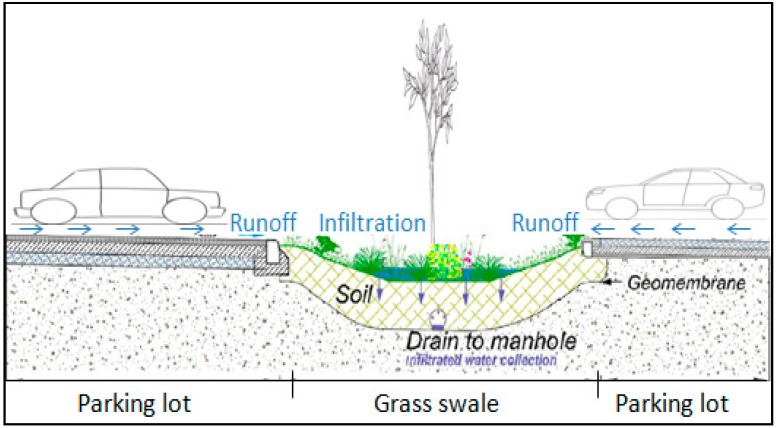
Cross section view of the grass swale system.

Figure 2 shows a cross section of the grass swale system. It also shows that grass swale has parking space on the both sides. It also shows the system has a vegetation layer that usually grows on the soil media. The topsoil used was classified per the USDA soil texture classification system as sandy loam, which consists of sand, silt and clay. The grass swale is designed with 0.35 m deep filter soil layer on the top of gravel layer and 0.6 m infiltration trench. The swale had a slope of less than 1.5%, which allows lateral input flow from the parking lot surface without disrupting flow into the swale system and collection into the underground trench through the grass swale. Runoff from both impervious areas of the parking lot of almost 300 m^2^ total surface is collected into the swale system, infiltrated and any extra runoff drains to the manhole. The grass swale soil characteristics and design information are described in Table 2 below. Grass swale system runoff outflows were measured during different real rain events.

### 3.2. Data Processing and Analysis 

**Figure 3:**
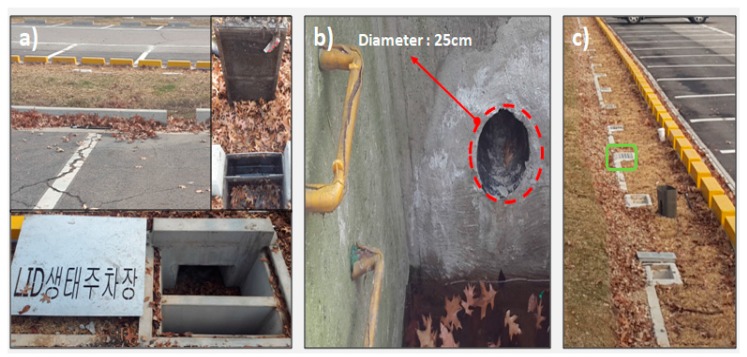
From left to right: (**a**) grass swale features with infiltration trench; (**b**) Drain water pipe of about diameter 25 cm where the infiltrated water outflow was measured and then water entered into the underground storage trench; (**c**) the location where the water level was measured.

The grass swale hydrological performance was investigated from May 2017 to October 2017. Continuous measurements of rainfall runoff retentions were taken to provide a comprehensive evaluation of the grass swale stormwater mitigation performance during real rain events in the urban parking lot of Mapu-gu, Seoul, Korea. Measured rainfall data were obtained from the Korea Meterological Administration (KMA) data site by selecting the nearest rain gauge, which is within 300 m distance from the site [38]. The uncertainty in the storm data collected from the KMA was found to be less than 12% during the analysis. Water flow, height and soil moisture values were measured at the different positions in the grass swale system: (1) water flow in the grass swale system during rain events; (2) water level in the grass swale system when water was flowing towards the underground trench during big rain events; (3) soil water contents were measured at the convex and concave surface where the water varies during the different time periods. The OTT Orpheus Mini Water Level Logger (OTT Hydromet GmbH, Kempten, Germany) is an integrated pressure sensor which was used for the surface water measurement in the grass swale during different time intervals. A Stingray 2.0 (Greyline instruments Inc., Largo, Florida, USA) portable level velocity logger was installed in the grass swale system to check the water flow of the grass swale during different storm events. Similarly, two venire soil moisture sensors were used in the grass swale system to check the soil water content during different time periods. Grass swale outflows were measured with the help of perforated pipe near the storage trench. Water flow and soil moisture contents also measured from the grass swale with the help of the abovementioned devices. Figure 3 shows the points of the grass swale system where the water enters and infiltrates into the trench. Under the soil of swale system, there was a 25 cm diameter porous pipe which was installed to collect the infiltrated water and lead it to the underground trench. All the infiltrated water is collected into the underground storage trench which consists of gravel with local native soil. The main purpose of the underground trench was to collect the infiltrated water and infiltrate it into the ground surface. The total depth of the underground trench was 0.6 m. Experiments were performed during real conditions for the longer period of around 6 months to check the hydrological performance of grass swale in Korean conditions.

## 4. Results 

Results included the soil water content, water level and flow of rainfall runoff measurements during the different storm events at the grass swale system.

### 4.1. Grass Swale Soil Moisture Response

**Figure 4:**
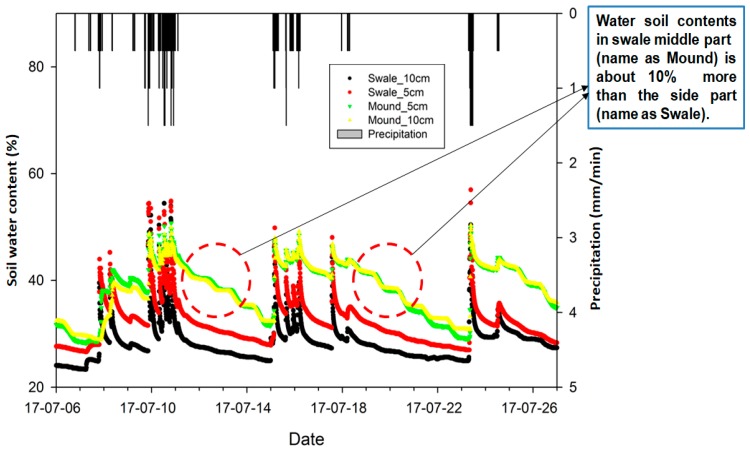
Show the precipitation (mm/min) and the soil water content (%) of the grass swale system at the middle part (named mound) and at the side part (named swale).

Over the 6-months monitoring period, soil moisture of the grass swale was investigated during different storm events varying from 50 mm/h to 100 mm/h, as shown in Figure 4. Soil water content was the dominant mechanism for runoff mitigation through the grass swale. Soil water contents were measured at different depths at two different points, as shown in Figure 1. The first point is referred as the swale (side part of swale system) where the soil water content was measured at 5 cm and 10 cm depth. The second point (which a rounded mass projecting above a surface of grass swale) is called the mound, and the soil moisture was also measured at 5 cm and 10 cm depth. Swale, mound and precipitation were shown in different colors in Figure 4 below. As the mound is the middle part which has a lower elevation as compared to swale part of the grass swale therefore runoff stayed in the mound part for a longer period of time than in the swale. As a result of this, the mound part soil also captured more rainwater as compared to the side part swale. Water content is the mound part is almost 10% higher than as compared to the swale part of the grass swale system, as manifested in Figure 4.

Soil moisture was measured in the grass swale system during rainfall events of different intensity, ranging from 20 mm/h to 100 mm/h, during 6–26 July 2017. The soil water contents of the swale system were increased as the intensity of rain event increased. This is because under the big rain events, soil can capture more rainwater as compared to the small rain events [24]. When the intensity of rainfall runoff was at the maximum value of about 100 mm/h, the soil water contact in grass system was also captured more rainwater and the maximum moisture content of soil was found to be around 57%, as shown in Figure 4. During the different storm events, overall soil water contents of the grass swale system varied from 35% to 57%. One of the advantages of grass swale soil water capture is that it can reduce the total surface runoff and also helps to increase the evapotranspiration rate [24,25], which can reduce the risk of flash flooding problems in the parking lot. 

#### Soil Moisture Seasonal Effect

**Figure 5:**
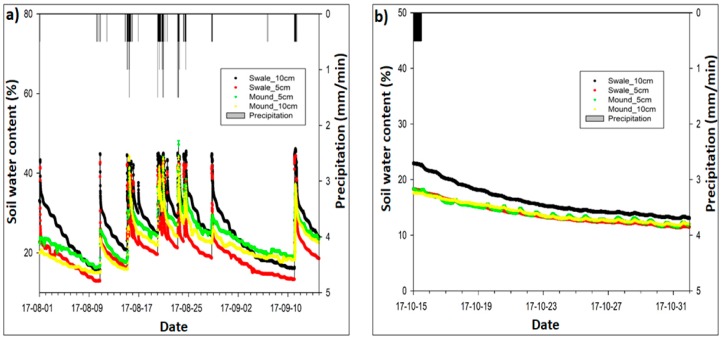
Shows the seasonal effect on the soil water content of the grass swale system. (**a**) soil water content of the grass swale in summer and (**b**) soil water content of the grass swale in summer in autumn seasons.

Seasonal effect on mean soil water content was found to be significant when all storm events were analyzed, as shown in Figure 5. Seasons show a distinctive set of climate patterns, which affect the soil moisture capacity of the grass swale soil and vegetation during different preceding wet and dry periods. Such preceding periods significantly affect the soil moisture of the grass swale. This study was carried in summer and autumn seasons (Figure 5a,b) to check the soil moisture of the swale system. Results indicated that the soil moisture of the summer season with relatively big storm events was higher as compared to the autumn with relatively small storm events. This finding proved that the rain events significantly attributed to the soil moisture value of the grass swale. Therefore, the grass swale rainfall runoff performance will be more effective in summer as compared to autumn. 

### 4.2. Grass Swale Water Level Measurement

**Figure 6:**
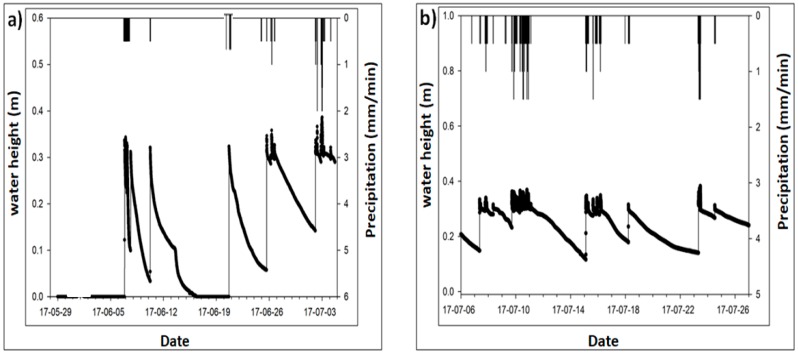
Shows the depth of the total amount of rainfall runoff which is attenuated in the grass swale during the different storm events of: (**a**) 29 May–3 July 2017; (**b**) 6–26 July 2017.

In the past, probability plots were used to evaluate the rainfall runoff attenuation [39] however, in recent times stormwater control measures (SCMs) [25] were employed to check the volume attenuation of grass swale. The grass swale system produces the similar volume attenuation in different storm events as shown in Figure 6. From the different storm events analysis results, the grass swale system vegetation and soil layers captured the rainfall runoff for a longer period of time. Then it water slowly infiltrated into the underground soil where the perforated pipe collected all infiltrated water and led it into the underground trench. Firstly, the rainfall runoff in retained and if the soil is fully saturated, then little volume attenuation was found, as shown in Figure 6. 

Water height was measured on the surface of the grass swale system as manifested in Figure 6. The maximum volume retention was found to be 0.4 m when there was a big rain event of about 110 mm/h intensity. Figure 6 also indicates that more volume was retained during big storm events of 110 mm/h as compared to small storm events of 50 mm/h. During the analysis, it was also found that there was no runoff outflow to the underground trench produced from the grass swale during small storm events. The rainwater first retains on the surface of grass swale and extra rainwater flows into the underground storage trench. The results also indicated that the grass swale can be used to capture a large amount of rainfall runoff, which helps to control flash flooding problems in Seoul, Korea.

### 4.3. Rainfall Runoff Attenuation

**Figure 7:**
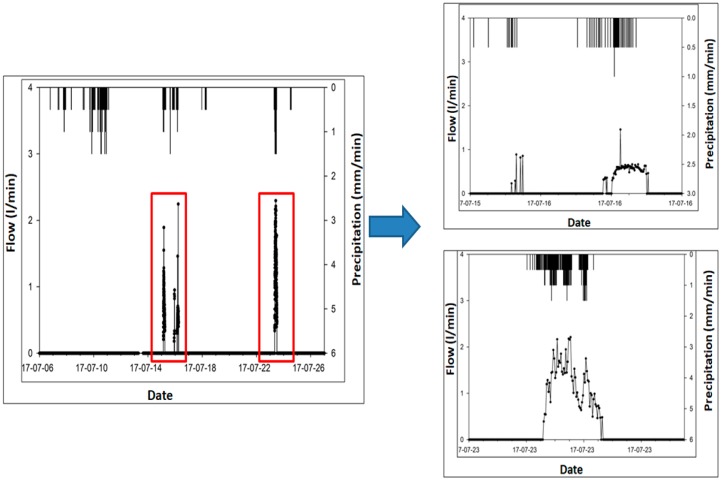
Indicates the rainfall runoff outflow (L/min) response during to the different storm events of 6–26 July 2017.

During the analysis of the grass swale, the storm events hydrograph data provides details of the grass swale’s response to the different rainfall runoff inputs. Hydrographs of different storm events were expressed by the capture of rainfall as shown in Figure 7. The hydrograph responses show fluctuations with different storm events. However, in a 60 mm/h first rain event, no discharge was found from the grass swale.

A 15 July 2017 storm event of 90 mm/h and the corresponding hygrograph response are shown in Figure 7. From the results, the runoff outflow to the trench was only about 1.0 L/min which helped control the surface runoff impact problems. This is the typical behaviour of the runoff management at the grass swale site shown below. From the analysis, it was proved that grass swale can capture around 40% to 75% of the rainfall runoff during small rain events of intensity about 30 mm/h. This is because the grass swale has the ability to store a large amount of rainfall runoff in its vegetation and soil layers. This process not only decreases the rainfall runoff volume, but also increases the concentration time.

**Figure 8:**
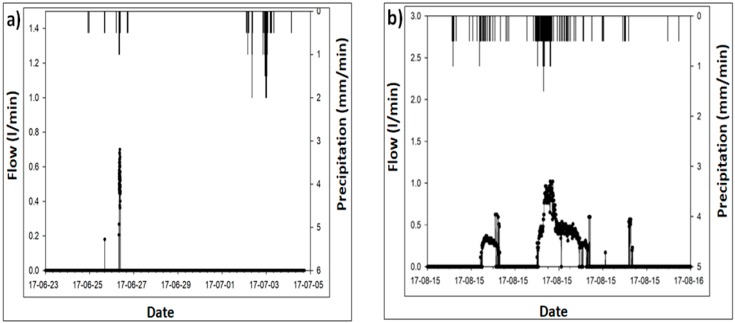
Rainfall runoff outflow (L/min) response during the different storm events: (**a**) 23 June–5 July 2017; (**b**) 15–16 August 2017.

Similarly, the hydrograph of 23 July 2017 shows the grass swale response against bigger storm events of 100 mm/h. The hydrograph indicates same characteristics found in moderate storm events, such as rainfall runoff and peak flow reduction. However, in this case, grass swale exhibited less reduction in peak flow reduction. The maximum runoff outflow response of grass swale to underground trench was 2.25 L/min, as shown in Figure 8. This also confirms that the additional runoff entered from the surrounding surfaces including impervious parking lots and road areas. Rainfall runoff entered from the all four sides (impervious parking lot) into the grass swale. Swale system captured all rainwater and gradually infiltrated it into the underground surface. 

Figure 8 also shows the runoff flow response from the grass swale system to underground trench system during various storm events of 17–June 2017–5 July 2017 and 15–16 August 2017. It shows that during the different storm events the runoff flow from the grass swale varied from 0.8 L/min to 1.1 L/min. From the analysis of the results, on average the total rainfall runoff retention on grass swale was found to be 40% to 75% during the small strom events of 30 mm/hr. This helps to not only attenuate the storm water for a longer period of time, but also to reduce the surface runoff in the urban parking lot of Mapu-gu, Seoul. Based on the research results, it is proved that grass swale is very helpful to capture a large amount of rainfall runoff for a longer period of time. 

## 5. Discussion

The main objective of this study was to verify that the grass swale system is a very useful technique for stormwater management in the Mapu-gu urban parking lot (Seoul, Korea). It can retain rainfall runoff for a longer duration and it can minimize the outflows to the underground storage trench during different storm events. Moreover, the results also indicated that the grass swale reduced the runoff volumes and peak runoff, delayed peak runoff and distributed rainfall runoff over a longer period of time. 

This experimental study reflects the previous research results [25] and proves that the grass swale is expected to provide momentous improvements in rainfall runoff retention performance over the impervious surface runoff management systems. The grass swale system is expected to capture over 40% of the rainfall runoff on an average during the different storm events. From the analysis of the field experiments, it was proved that grass swale may retain total runoff about 40–75% of small storm events. 

From the analysis, grass swale runoff volume reduction results were found to be higher than those reported by Barrett [20], Deletic [23] Rujner et al. [24] and Rushton [22]. Therefore, this grass swale is very helpful for urban runoff management. This is because in the grass swale system first rainwater is detained in the vegetation and soil media, infiltrated rainwater is collected with the help of a perforated pipe and then passes into an underground storage trench where water is stored and infiltrated into the underground soil. This study stressed on the application of grass swale in an urban parking lot because the grass swale can help to change the impervious surface to a green area which not only captures a large amount of rainfall runoff but also increases the time of concentration.

This experimental study has investigated the grass swale performance for storm water management during various rain events. However, one of the limitations of this study was that the grass swale runoff management performance was only monitored for a short period of time during the summer and autumn seasons. There is a high need for long term monitoring in the summer as well as the winter seasons. This will provide more accurate analysis results of its performance in storm water management over a longer period. This study used the native soil (loamy sand) for the retention of rainwater. The soil is a very important factor [21,24,25] during the retention and infiltration of different rain events. Further work is required to examine the different soils as well as vegetations performance of the grass swale system at the real site. This could help to control rainfall runoff quantity and quality in urban areas. In the future, there is also a high need to combine the grass swale system with other suitable LID practices for optimal and multi-functional benefits for sustainable storm water management in urban areas. For example, Brasswell et al. [40] noted that if permeable pavement is connected in series with biofilters, it could reduce the rainfall runoff volume as well as improve the water quality in urban areas. Therefore, in future there is a high need to think of the optimal utilization of public space for LID applications to make our cities sustainable and more resilient to city climate change.

## 6. Conclusions

A grass swale installed in the parking lot of a World Cup stadium was designed to check the performance during real storm events. Swale discharge, moisture contents and rainfall runoff flow were measured to evaluate the grass swale system for storm water management in a highly urbanized area of Seoul, Korea. Storm events were monitored and analyzed under real climatic conditions of Seoul, Korea. This field study provided some useful findings, as explained below:

○ Grass swales are very effective during small rain events because all the rainwater infiltrates into the swale soil and thereby was zero rainfall runoff outflow during these small events. The grass swale construction tries to mimic the natural hydrological conditions by changing concrete pavement parking into natural vegetation. Grass swale vegetation not only captures rainfall runoff but also evaporates water into the atmosphere. During big storm events, the results indicated that the grass swale soil captured more rainwater and hence enhanced the moisture content from 35% to 57%. It was also found that the selection of soil media is very important for grass swale construction [24,25]. As the grass swale has three specific functions: retention, infiltration and conveyance. However the first two segments—retention and infiltration—are very important factors that can hold and infiltrate all rainfall runoff and produce zero runoff. During this study, the grass swale retained rainfall runoff from around 0.3 to 0.4 m rainfall during different storm events. From the field experiments, it was proved that grass swale may retain a total rainfall runoff of about 40–75% of small storm events.
○ It is also proved that during small storm events the hydrographic peak is much smaller as compared to big storm events. This is due to the fact that in the case of small storm events almost all the rainwater is infiltrated and captured in the grass swale. However, in case of big storm events as the soil depth of grass already fully saturated and does not have the ability to capture more runoff, therefore the runoff flows into the underground trench. However, future studies need to be carried out by increasing the soil characteristics for more runoff retention performance of the grass swale and thereby checking their performance for stormwater management in urban areas. All the results provided in this study proved that the grass swale is a very effective best management practice to control rainfall runoff in highly urbanized areas like Seoul, Korea.

## Figures and Tables

**Table 1:** Rainfall runoff volume reduction of different grass swale system.

Reference	Runoff Volume Reduction (%)	Location
Deletic and Fletcher (2006) [18]	33–87%	Brisbane, Australia
Ackerman and Stein (2007) [19]	52.5%	Los Angeles, USA
Barrett (2008) [20]	50%	USA
Fassman and Liao (2009) [21]	63.7%	New Zealand
Rushton (2001) [22]	30%	Florida, USA
Deletic (2001) [23]	45.7%	University of Aberdeen, UK
Rujner et al.(2016) [24]	40–55%	Luleå, Sweden
Davis. et al. (2012) [25]	59%	Maryland, USA

**Table 2:** Design characteristics of the grass swale system.

Parameter	Characteristics of Grass Swale
Runoff source ^a^	Parking lot for around 30 cars
Design details	0.35 m deep filter soil layer on the top of gravel layer and 0.6 m infiltration trench
Filter soil composition	Local soil mixed with 1:1 with sand in grain size 0.02–2 mm
Grass swale side slope	<2.5%
Surrounded impervious areas (runoff entering from all this area to grass swale)	Around 300 m^2^
Catchment and infiltration area ratio	4:1
Grass swale length	51 m
Grass swale width	1.2 m
Soil compositions	Made up of using local soil composition (sand + silt + clay)
Monitoring period	May 2017–October 2017

^a^ 100% impervious asphalt pavement.
